# Visceral Leishmaniasis in Muzaffarpur District, Bihar, India from 1990 to 2008

**DOI:** 10.1371/journal.pone.0014751

**Published:** 2011-03-04

**Authors:** Paritosh Malaviya, Albert Picado, Shri Prakash Singh, Epco Hasker, Rudra Pratap Singh, Marleen Boelaert, Shyam Sundar

**Affiliations:** 1 Institute of Medical Sciences, Banaras Hindu University, Varanasi, India; 2 Epidemiology and Disease Control Unit, Department of Public Health, Institute of Tropical Medicine, Antwerp, Belgium; The George Washington University Medical Center, United States of America

## Abstract

**Background:**

Visceral Leishmaniasis (VL) is a vector-borne disease transmitted by *Phlebotomus argentipes*. To understand the VL seasonality, annual and monthly variations of VL incidence and its relationship to meteorological variables, the numbers of VL cases reported in Muzaffarpur district, Bihar, India from 1990 to 2008 were studied.

**Methods:**

Annual VL incidence per 10,000 and the total number of annual VL cases reported at block Community Health Centres (CHC), Public Hospitals or Non-Governmental Organisations (NGO) and the number of VL cases per month from 2000 to 2008 as well as the monthly average of cases for 2000–08, 2000–04 and 2005–08 periods along with the monthly averages of temperature, rainfall and relative humidity were plotted. VL Standardised Incidence Ratios per block were computed for the periods of 1990–1993, 1994–1998, 1999–2004 and 2005–2008 and month wise from 2002 to 2008. A negative binomial regression model was used to evaluate the association between meteorological variables and the number of VL cases per month from 2000 to 2008.

**Results:**

A total of 68,358 VL cases were reported in Muzaffarpur district from 1990 to 2008, ranging from 1,2481 in 1992 to 1,161 in 2001. The blocks with the highest number of cases shifted from East (1990–98) to West (1999–2008). Monthly averages of cases ranged from 149 to 309, highest peak in March–April and another one in July. Monthly VL incidence was associated positively to rainfall and negatively to relative humidity and the numbers of VL cases in the previous month.

**Interpretation:**

The number of cases reported to the public health sector allowed the describing of the spatial distribution and temporal variations in the Muzaffarpur from 1990 to 2008. However, to assess the actual VL burden, as well as the efficacy of the control measures applied in the district, reporting from private practices and NGOs should be encouraged.

## Introduction

Leishmaniasis is a group of vector-borne diseases caused by *Leishmania* genus parasites and transmitted by Phlebotomine sand flies. Leishmania organisms are endemic in more than 80 countries and 350 million people are considered to be at risk [1]. Leishmaniasis has three clinical forms: visceral, cutaneous and mucocutaneous of which visceral leishmaniasis (VL) is the most severe form and is fatal if untreated. In the Indian subcontinent VL, also known as kala-azar, is caused by *L. donovani* transmitted by *P. argentipes* in an anthroponotic cycle [2]. Bihar state contributes 50% of the VL caseload in the subcontinent and 90% in India [3]. Since 2005, VL endemic countries in the Indian subcontinent have reinforced their commitment to eliminate VL from the region by 2015. The target is to reduce the annual VL incidence to less than one new case per 10,000 populations [4]. In India, the National Kala-azar elimination program is based on vector control – Indoor Residual Spraying (IRS) of houses and cattle sheds – and early detection and treatment of cases in VL endemic districts. The latter relies on the public primary health care system at district and block (district subdivision) levels [5] and it is monitored using passive surveillance. Under the national program, all the public health facilities and optionally the Non-governmental Organisations treating VL patients report the number of patients treated per month to the state health authorities through district hospital. Even if the reported figures are an underestimation of the real burden of VL [6], those statistics have been used to describe the disease dynamics and to monitor the impact of control measures [7]. The fluctuation of the number of VL reported cases in the last 30 years in VL endemic regions in India can be linked to variations in “herd immunity” or in effectiveness of control (e.g. emerging DDT resistance and resistance to sodium stibogluconate). However VL, as any vector borne disease, is also influenced by meteorological and environmental conditions. In the Indian subcontinent, temperature and humidity regulate the development of *P. argentipes* [8]. Meteorological factors (i.e. temperature, rainfall) and environmental factors (i.e. soil temperature and moisture) have been associated to *P. argentipes* monthly abundance in Bihar [9] and West Bengal [10] respectively. Similarly, an environmental study using remote sensing found an association between VL incidence and air temperature (25.0–27.5°C), relative humidity (66%–75%) and annual rainfall (100–160 cm) in the Gangetic plain [11].

In this paper we described the number of VL cases reported in Muzaffarpur district at block and district levels from 1990 to 2008. Standardised incidence ratios (SIR) were used to study annual variations at block level from 1990 to 2008 and monthly variations from 2002 to 2008. Monthly reported cases from 2000 to 2008 were used to study VL seasonality and its relationship to basic meteorological variables (i.e. temperature, rain and relative humidity).

## Materials and Methods

### Study area

The study was conducted in Muzaffarpur district in Bihar (26.07°N 85.45°E, area 3175 km^2^). Average temperatures vary from 32°C in April–May to 14°C in December–January. Rainfall is also variable with a rainy season from June to September. Muzaffarpur is endemic for VL; cases have been reported since 1972 and accounts for over 70,000 reported cases in the past two decades. The district has 14 blocks with an average of 300,000 inhabitants per block and geographical areas ranging from 139 km^2^ (Musahari) to 282 km^2^ (Paroo). There are 14 Community Health Centres (CHC; erstwhile Primary Health Centres) – one per block, one district hospital and one medical college reporting the monthly number of VL cases to the district headquarter in Muzaffarpur city [5]. Some NGOs treating VL patients also report the number of cases to the district headquarter.

### Data sources

The total number of VL cases reported per year in Muzaffarpur district and information on their origin (i.e. CHC, hospital or NGOs clinic) between 1990 and 2008 were obtained from the Ministry of Health district headquarters in Muzaffarpur. Month-wise distribution of VL cases in the district was available only from 2000 to 2008. The numbers of VL cases per month reported to each CHC were available from 2002 to 2008. CHCs and public hospitals record the number of VL patients treated in paper forms. A monthly report is forwarded to the ministry of health district headquarter where a joint accumulative report is prepared. There were no obvious missing values in the reporting by the public sector (i.e. VL cases reported from public hospital and CHCs every year) but reporting by NGOs was irregular (i.e. no cases reported from 1998 to 2004). From 1990 to 2008 there were no changes in the number of public health facilities treating VL patients in Muzaffarpur district. However, changes in the private sector are difficult to assess.

VL diagnosis was similar over the study period. According to the guidelines, patients presenting chronic fever, loss of appetite, weight loss, skin pigmentation and abdominal distension were considered VL suspects. After clinical exploration to determine splenomegaly and discard other pathologies VL cases were confirmed by serological tests i.e. aldehyde test or rK39 dipstick. rK39 disptick was available from 2002 in private sector and from 2006 in public sector (CHCs and public hospitals). Relapse, recurrent or complicated cases in CHCs were referred to public hospitals for microscopic parasitological examination of splenic aspirates.

The yearly population in Muzaffarpur district and blocks was extrapolated from the 2001 census data assuming a yearly population growth rate of 2.674% [12]. Meteorological variables: monthly rainfall (mm), average temperature (°C) and relative humidity (%) from January 2000 to December 2008 were obtained from the Indian Meteorological Department's station in PUSA institute with its unit in Samastipur district which is 35 km away from Muzaffarpur city.

### Analyses

#### Descriptive

The total number of VL cases reported per year and their origin (i.e. proportion of reported cases from CHCs, district hospitals, medical college and NGOs) as well as the annual VL incidence per 10,000 people were plotted. The number of VL cases per month from January 2000 to December 2008 were plotted to describe the VL monthly dynamics. The seasonality was assessed by plotting the average number of cases per month for 2000–08, 2000–04 and 2005–08 periods. The monthly averages of mean temperature, mean rainfall and mean relative humidity were also plotted.

#### Standardised Incidence Ratios

**Figure 1:**
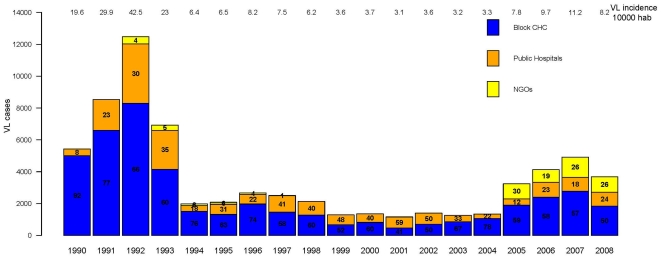
The total numbers of Visceral Leishmaniasis (VL) cases reported in Muzaffapur district per year from 1990 to 2008. The proportion of cases reported to the different health care facilities: Community Health Centre (CHC), Public Hospitals or Non-Governmental Organisation (NGO) is represented as percentage of the total cases per year inside boxes of different colours. The annual VL incidence rate (per 10,000) in Muzaffarpur district is noted at top of the figure.

To study the spatial distribution of reported cases in the district from 1990 to 2008, VL Standardised Incidence Ratios (SIR) were computed per block and for the following periods (based on VL incidences, figure 1): 1990–1993, 1994–1998, 1999–2004 and 2005–2008 using the VL incidence per period in all blocks as reference [13]. SIR is a relative measure that allows examining the spatial distribution of reported cases across time periods. It is calculated as the quotient of the observed and the expected number of cases multiplied by 100. A SIR greater than 100 indicate that more VL cases were reported than expected in that block i.e. a SIR of 180 corresponds to 80% more cases than the expected. Similarly, to assess the yearly distribution of reported cases from 2002 to 2008, SIRs were calculated per month using the VL incidence per month in all blocks as reference. The results were represented as chloropleth maps. Additional VL SIR Maps (i.e. results per year and per month from 2002–04 and 2005–08) are available as additional material (Figure S1, Figure S2, and Figure S3). SIR and maps were produced using spdep in R 2.10.1 (www.r-project.org/).

#### Meteorological Modelling

A negative binomial regression was applied to study the VL dynamics in Muzaffarpur. First, univariate analyses were used to determine the time lags that maximised the association between meteorological variables (i.e. monthly average temperature (°C), relative humidity (%) and total rain fall (metres)) and monthly cases. Variables with a P-values≤0.10 were incorporated in a multivariate model. The initial model also included (1) “year” as a fixed effect to adjust for yearly variation and (2) the number of cases in the previous month to adjust for autocorrelation. The final negative binomial model was obtained by backward selection using a P-value≤0.05 as the criterion. A variable with the total precipitation in the previous year (i.e. total metres of rainfall over 12 months) was added to the final model and kept if the model fit was improved. The yearly estimated population was used as exposure and the results were presented as Incidence Rate Ratio (IRR). Robust standard errors were used. The average number of cases per month estimated by the model was calculated. Fitted values were plotted and the residuals were evaluated using the average percent error (considering positive and negative errors equally) as indicator. All statistical analyses were conducted using Stata 11 (Stata Corporation, College Station, TX).

## Results

The number of VL cases reported per year in Muzaffarpur district ranged from 12,481 (VL incidence rate 42.5/10,000) in 1992 to 1161 (VL incidence rate 3.1/10,000) in 2001. Four phases could be discriminated: 1990–93 (average annual VL incidence rate 23.2/10,000, 95% CI 22.6–23.8), 1994–98 (average annual VL incidence rate 5.2/10,000, 95% CI 4.9–5.4), 1999–2004 (average annual VL incidence rate 2.2/10,000, 95% CI 2.1–2.4) and 2005–2008 (average annual VL incidence 5.8/10,000, 95% CI 5.5–6.0). The proportion of cases reported to by the block CHCs, public hospitals and NGOs varied over the study period. Block CHCs were consistently the health facilities reporting the larger number of VL cases except for 2001, the year with the lowest VL incidence, when 59% of the cases were reported from public hospitals. The number of cases reported by NGOs increased from 2005 (figure 1).

**Figure 2:**
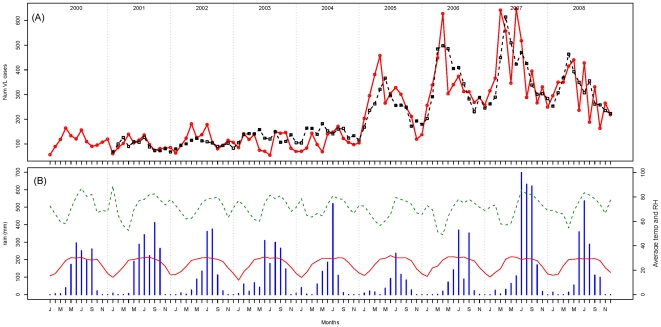
Observed and predicted VL cases and recorded meteorological parameters for each month from 2000 to 2008. (A) The observed (solid red line) and the predicted (dashed line – estimated from the negative binomial model) numbers of monthly Visceral Leishmaniasis (VL) cases in Muzaffarpur district from 2000 to 2008. (B) Monthly rainfall in mm (blue histogram), average temperature (temp) in °C (solid red line) and average relative humidity (RH) in % (dashed green line).

**Figure 3:**
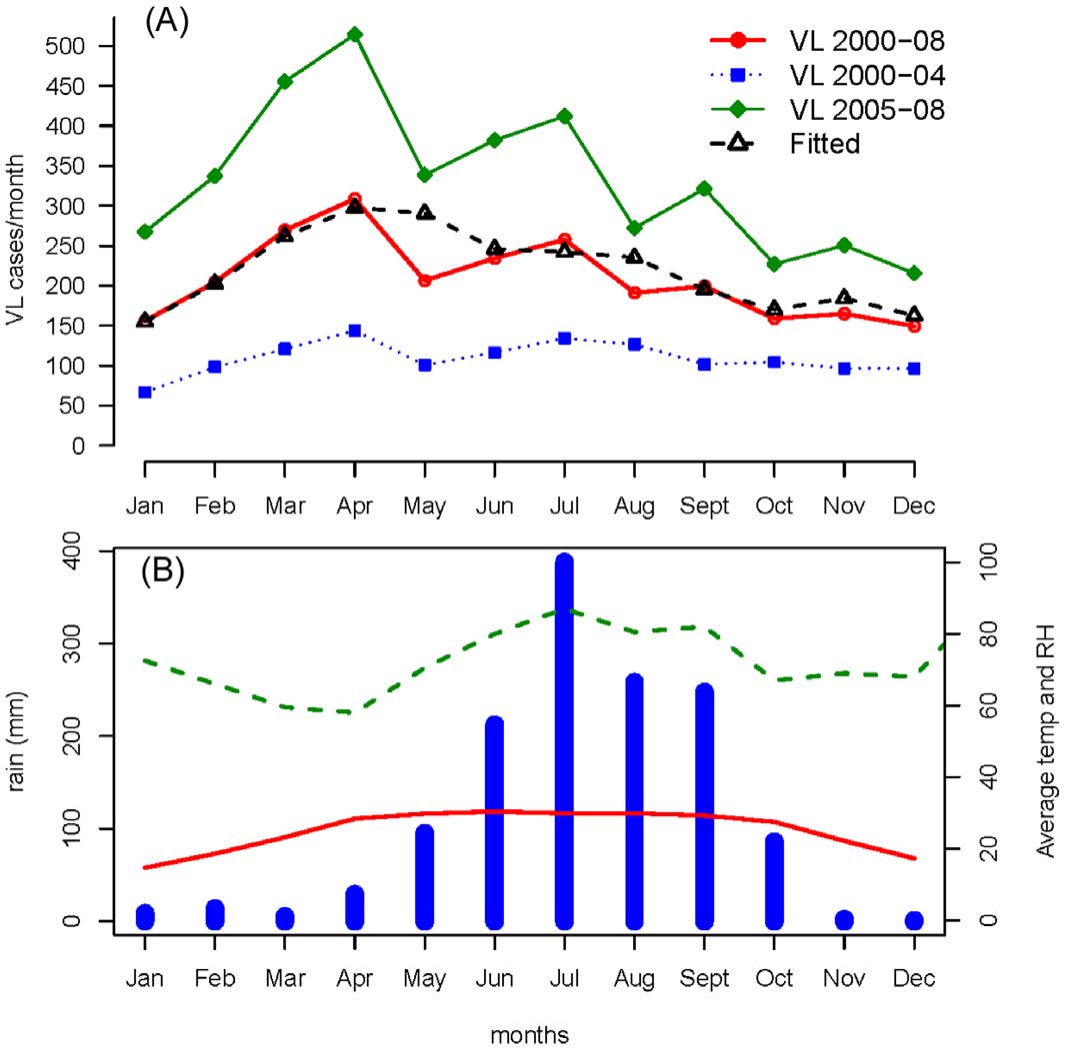
Seasonality of VL cases in the two phases (2000–04 and 2005–08) identified during the study period. (A) The observed average number of monthly Visceral Leishmaniasis (VL) cases in Muzaffarpur district: from 2000 to 2008 (solid red line), 2000 to 2004 (dotted blue line) and 2005 to 2008 (green line). Predicted average of monthly VL cases from 2000 to 2008 (estimated from the negative binomial model) plotted as black dotted line. (B) Monthly average rainfall in mm (blue histogram), temperature (temp) in °C (red line) and relative humidity (RH) in % (dashed green line).

Two distinct periods were evident when monthly reported cases from 2000 to 2008 were plotted: 2000–04 with 6535 VL cases (annual average 1307) and 2005–08 with 15973 registered cases (annual average 3993) (Figure 2). Monthly averages of reported VL cases ranged from 149 to 309, but there was a clear seasonality with a peak of cases in March–April and possibly a second minor increase in July (Figure 3). The seasonality was similar in the two phases identified during the study period (i.e. 2000–04 and 2005–08) (Figure 3). The April peak corresponded to the hottest period of the year with the lowest relative humidity in contrast to July which was the rainiest month (Figure 3).

**Figure 4:**
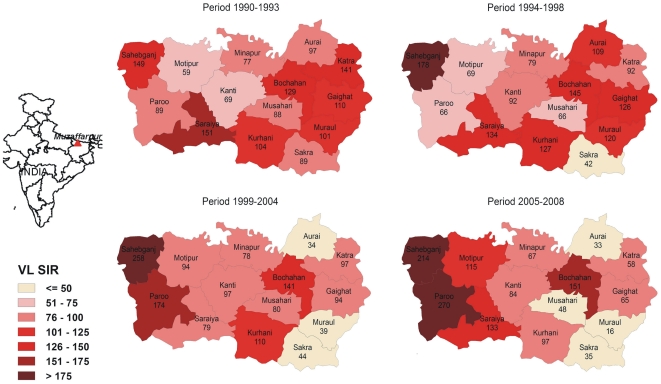
Visceral Leishmaniasis (VL) Standardised Incidence Ratio (SIR) per block in Muzaffarpur district in Bihar, India. VL SIR computed for the following periods: 1990–1993, 1994–1998, 1999–2004 and 2005–2008 using the VL incidence per period in all blocks as reference.

**Figure 5:**
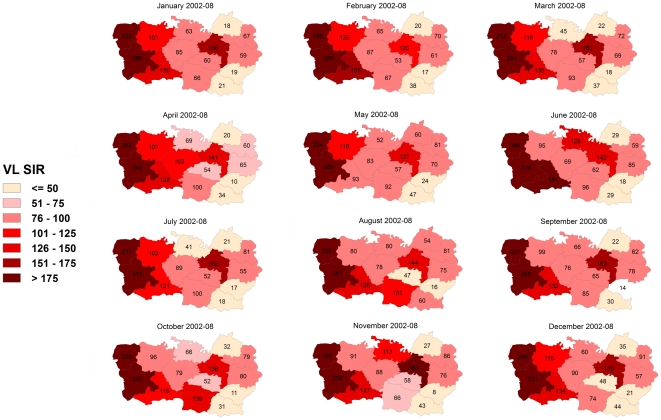
Visceral Leishmaniasis (VL) Standardised Incidence Ratio (SIR) per block and month in Muzzafarpur district in Bihar, India from 2002 to 2008. SIR was calculated per month using the VL incidence per month in all blocks as reference.

The spatial distribution of reported cases in the 4 study periods from 1990 to 2008 illustrated in Figure 4 shows that some blocks had constantly higher (Sahebganj and Bochahan) or lower (Sakra) SIR over time. Taking the border between Kanti and Bochahan, Musahari and Kurhani blocks as a reference to divide the district, the blocks with the highest number of cases were mainly located in the East in 1990–93 and 1994–98 periods. In both periods, 5 out of 8 blocks in the East had SIR>100 compared to 2 out of 6 blocks in the West. In the following period (1999–2004), the number of blocks with SIR>100 was unaltered in the West (2/6) but reduced in the East (2/8). In the last period (2005–08) the blocks with an excess of VL cases (SIR>100) were predominant in the West (4/6) and a minority in the East (1/8). No clear seasonality was observed when the distribution of VL cases was assessed per month from 2000 to 2008. Western blocks registered the higher number of cases every month and no big shifts (i.e. blocks going from very low to very high SIR) were observed (Figure 5).

The results of the negative binomial model show that monthly VL incidence was associated to meteorological variables: positively to rainfall lagged by 2 months and negatively to relative humidity of the same month and at lag 3 months and total rainfall in the previous year (Table 1). The number of VL cases in the previous month and the linear effect of year were also statistically significant and kept in the final model (Table 1). The model was able to identify April as the annual peak for VL cases (Figure 3) and had an average percent error of 25.2%.

**Table 1:** Final negative binomial model for monthly count of reported Visceral Leishmaniaisis (VL) cases in Muzaffarpur district from 2000 to 2008.

*Exploratory Variable*	*IRR*	*z*	*P-value*	*95% CI*
Relative humidity same month	0.98	−4.96	<0.001	0.97–0.98
Monthly relative humidity lagged by 3 months	0.98	−5.48	<0.001	0.97–0.98
Monthly rainfall lagged by 2 months	1.69	3.26	0.001	1.23–2.32
Total rainfall in the previous year	0.87	−2.49	0.013	0.78–0.97
Linear effect of year	1.14	7.99	<0.001	1.12–1.18
VL cases reported in the previous month	1.0012	3.33	0.001	1.0004–1.0019

Results presented as Incidence Rate Ratio (IRR).

## Discussion

The trend of VL cases in Muzaffarpur district from 1990 to 2008 (Figure 1) is similar to the trend observed in the whole Bihar state [7]. In Bihar, 58000 VL cases were reported per year from 1990 to 1993. This figure was reduced in the following periods: 1994–98 (less than 20000 cases/year) and 1999–2004 (around 12000 cases/year). In 2005 and 2006 the annual average increased over 25000 cases [7]. Muzaffarpur district seems to be a good model to evaluate the situation in the whole state. This is the first time that historical data of 19 years on VL incidence from Muzaffarpur have been analysed in detail. The reduction of cases after 1992 has been attributed by other authors to the implementation of two annual IRS rounds in the region [14], [15]. VL incidence rate increased again from 2005 onwards (Figures 1 and 2). This sudden increase may be due to an actual rise of VL cases in the district related to a rise in *L. donovani* transmission caused by the augment of *P. argentipes* (i.e. DDT resistance) or rise of populations' susceptibility (i.e. antimony resistance, higher HIV prevalence). However this phenomenon may simply reflect an increase of the proportion of reported cases linked to the launch of the VL elimination initiative and an intensification of control efforts in 2005. The access to public health facilities for VL treatment was promoted since 2006 when monetary incentives were provided to patients attending CHCs and public hospitals in Bihar. Noteworthy, the underreporting of VL decreased from 2003 to 2006 in Bihar [16] and the number of cases reported by NGOs increased during this period (Figure 1). The conclusion that the increase in VL after 2005 is due to reporting bias would also be supported by the fact that spatial distribution of reported cases in 1999–2004 and 2005–08 periods was similar (Figure 4).

The number of VL cases reported at block level seems to accurately reflect the temporal trend of VL in the district (Figure 1). The fact that block CHCs consistently report a higher proportion of VL cases compared to public hospitals indicates that these facilities are key in the VL case management in Muzaffarpur and their capacities to diagnose and treat VL should be strengthened. Similarly, the increase in the number of VL cases reported by NGOs from 2005 denotes the importance and capacity of these entities and the need to involve them in the VL elimination efforts. The shift in the spatial distribution of cases from West to East between 1990–1998 and 1999–2008 (Figure 4) may correspond to a rise in herd immunity in western blocks. Nonetheless, except some rare occasions annual VL incidences in each block (results not shown here) were consistently above the elimination target set by the regional governments i.e. to reduce the number of VL cases below 1/10,000 by 2015 [4].

The results of the regression model showed that monthly counts of VL cases were associated to meteorological variables. Those results should however be interpreted with caution. The meteorological variables in the model do not directly explain the number of VL cases reported per month as rainfall and relative humidity are not directly associated with the development of VL clinical signs but to *P. argentipes* density and seasonality as already shown in previous studies [9], [10], [17], [18]. Nevertheless, a rather simple model was able to accurately represent the disease dynamics and yearly seasonality of VL from 2000 to 2008 with a low average percent error (25%) compared to similar studies in malaria (50 to 68%) [19].

The VL seasonality is certainly linked to *P. argentipes* annual variations. However other factors may explain why VL reporting and *P. argentipes* peaks – April–July and May–November [9] respectively – are not symmetric. First, the highest *L. donovani* transmission periods have not been properly identified and may not correspond to *P. argentipes* peaks. The incubation period of *L. donovani* is not well established but it is supposed to range between 2 and 6 months [20]. Finally, treatment seeking behaviour may vary among individuals, social classes and period of the year. The temporal variations seem to be equally distributed across the district as there were not major changes in the distribution of reported VL cases per month from 2002 to 2008 (Figure 5). Nevertheless, as VL cases are reporting regularly to public health facilities, local health authorities should ensure for adequate supply of diagnostic tests and VL drugs throughout the year [5].

The VL data analysed in this paper correspond to cases treated in public health facilities and some NGOs in Muzaffarpur. These figures have been proven to underestimate of the real number of VL cases [6]. Including data from private clinics would have increased the number of cases but may have a limited impact on the seasonality and annual variations described here. Unfortunately the number of cases treated by private practitioners was not available. The underreporting decreased from 8 to 4 times between 2003 and 2006 [16] but the real number of cases is difficult to estimate as the level of underreporting may have varied over the study period. Similarly, the data reported to the health authorities was not detailed enough to assess their quality (i.e. duplicate cases), conduct subgroup analyses (i.e. new cases or relapse or defaulter) or evaluate other important aspects related to VL (i.e. HIV co-infection, PKDL). No quality control systems were in place to evaluate the completeness of the data collected or the quality of the case assessment, treatment outcome or data management. These are some of the limitations of the data used in this study, which are also used to evaluate its impact of the efforts to control VL in Muzaffarpur.

The recent decline in the number of VL cases reported in Muzaffarpur district from 2007 (n = 4920) to 2008 (n = 3679) and 2009 (n = 2355 – not analysed in this study) is encouraging. This drop may be related to the local and regional efforts to eliminate VL in general and to the use of improved diagnostic tools (i.e. rK39 dipstick) and anti-leishmania drugs (i.e. miltefosine) in particular. Despite this improvement, efforts should continue to ensure the accurate reporting of VL cases from public and private health facilities as well to get a clear picture of the situation. Active surveillance for early case detection, diagnosis and treatment and post treatment follow-up is the need of the time as VL case load is still way above the target to eliminate VL from the region by 2015. The reporting system should be improved so VL incidence per age, gender or population at risk groups (i.e. HIV, tuberculosis) as well as PKDL cases can be monitored.

## Supporting Information

Figure S1 SIR year wise from 1990–2008
(0.75 MB JPG)

Figure S2 Month wise SIR 2002–2004
(0.59 MB JPG)

Figure S3 Month wise SIR 2005–2008
(0.59 MB JPG)
