# Supplementary figures and images for: Visceral Leishmaniasis in Muzaffarpur District, Bihar, India from 1990 to 2008
Source: PLoS One. 2011 Mar 4;6(3):e14751. doi: 10.1371/journal.pone.0014751 (PMC3048857; doi:10.1371/journal.pone.0014751)

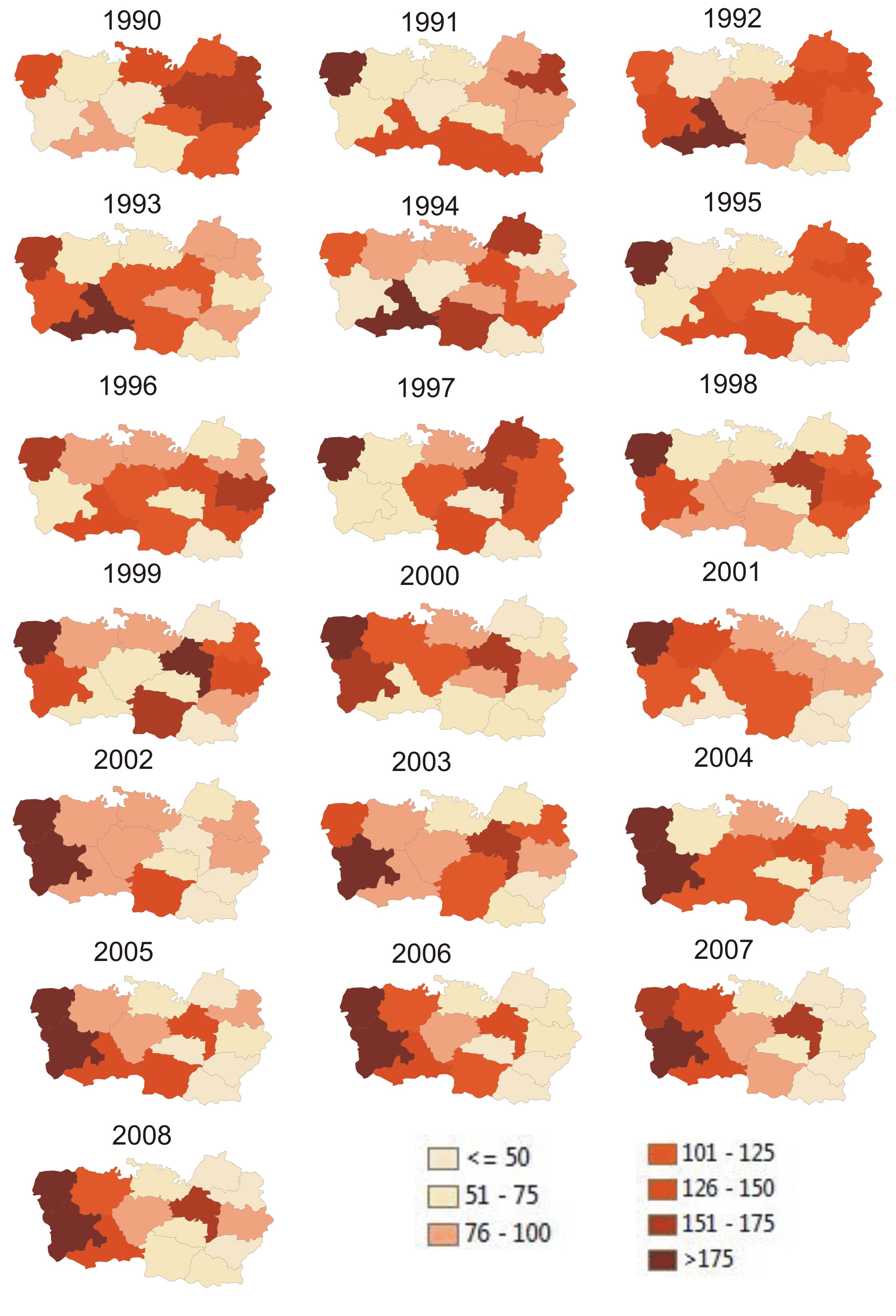

Supplement: Figure S1 — SIR year wise from 1990–2008 (0.75 MB JPG) [file pone.0014751.s001.jpg]

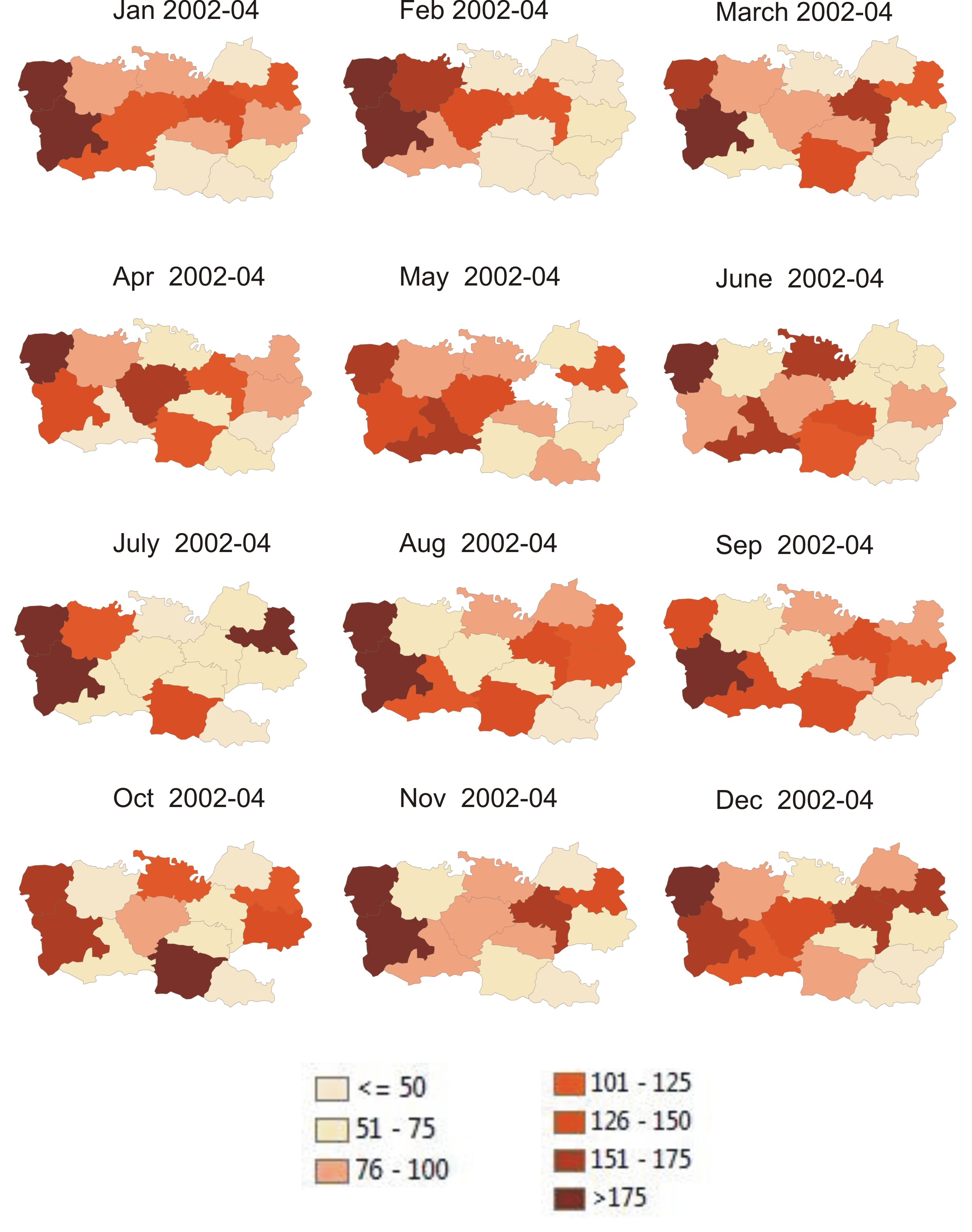

Supplement: Figure S2 — Month wise SIR 2002–2004 (0.59 MB JPG) [file pone.0014751.s002.jpg]

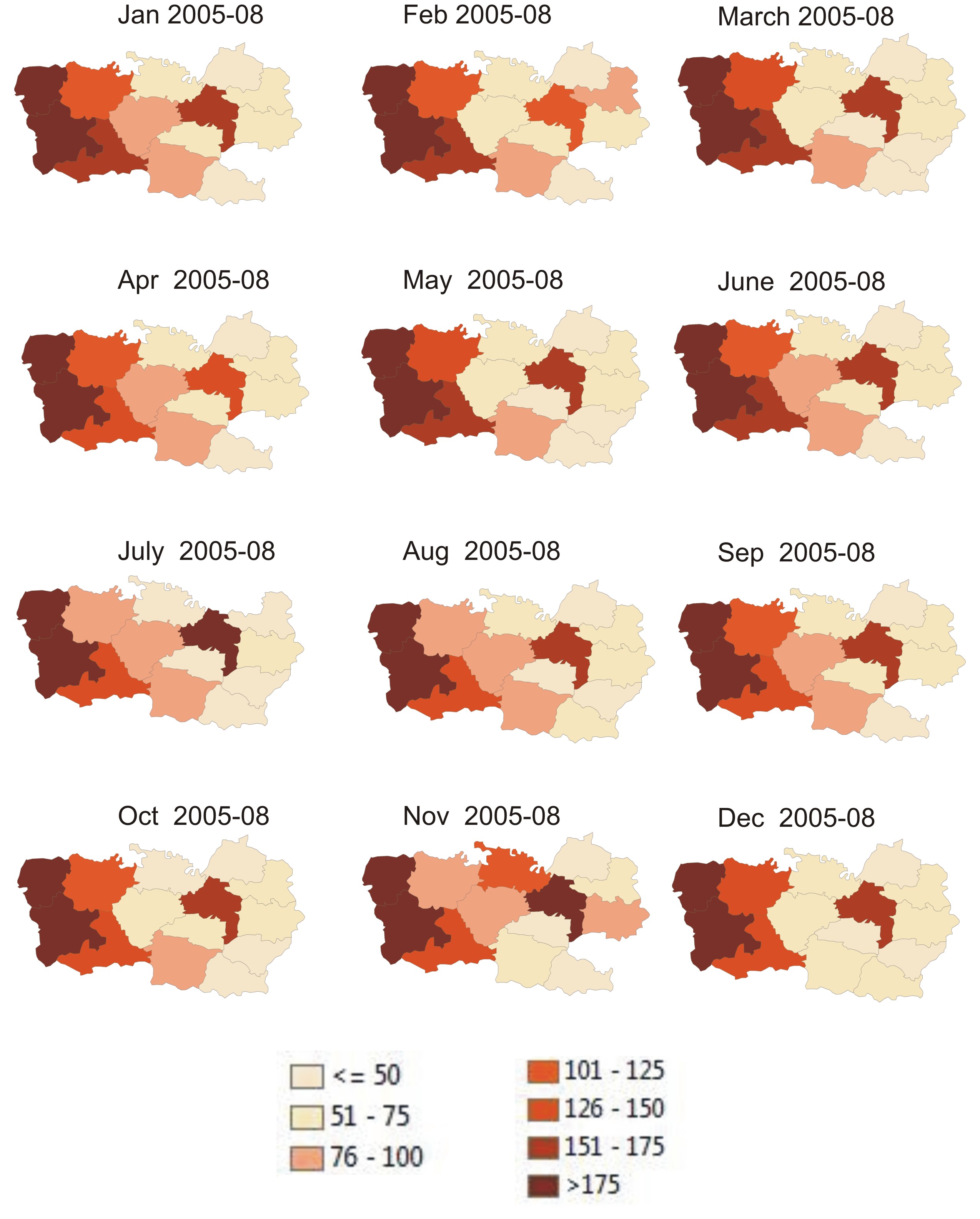

Supplement: Figure S3 — Month wise SIR 2005–2008 (0.59 MB JPG) [file pone.0014751.s003.jpg]
